# Experimental Investigation of the High-Temperature Rheological and Aging Resistance Properties of Activated Crumb Rubber Powder/SBS Composite-Modified Asphalt

**DOI:** 10.3390/polym14091905

**Published:** 2022-05-06

**Authors:** Zhizhong Zhao, Longlin Wang, Wensheng Wang, Xuanhao Shangguan

**Affiliations:** 1 Guangxi Hetian Expressway Co., Ltd., Nanning 530029, China; liuzyjlu2019@163.com; 2 School of Civil Engineering, Southeast University, Nanjing 211189, China; 3 Bridge Engineering Research Institute, Guangxi Transportation Science and Technology Group Co., Ltd., Nanning 530007, China; 4 Department of Road and Bridge, College of Transportation, Jilin University, Changchun 130025, China; sgxh1719@mails.jlu.edu.cn

**Keywords:** asphalt, activated crumb rubber, SBS, short-term aging, physical properties, rheological property

## Abstract

Crumb rubber could form the active groups on the surface by interrupting the crosslinking bond to improve the compatibility with asphalt. While styrene-butadiene-styrene block copolymer (SBS)-modified asphalt has excellent comprehensive properties, it has poor anti-aging performance and a high cost. To explore the influence of composite modification of activated crumb rubber powder (ACR) and SBS on asphalt, modified asphalt samples with different modifiers and SBS contents were prepared. Conventional physical properties tests, a dynamic shear rheometer (DSR), and the thin-film oven test (TFOT) were used to study the conventional physical properties, high-temperature rheological properties, and aging resistance of asphalt. In addition, the action forms and distribution of modifiers in asphalt were observed by an optical microscope to characterize the micro-morphology of ACR/SBS composite-modified asphalt. Test results showed that after adding SBS, the softening point, ductility, and elastic recovery of ACR/SBS asphalt could be significantly improved, but the viscosity and softening point difference were also larger. At the same time, according to the complex shear modulus, phase angle, and rutting factor, SBS can effectively improve the high-temperature deformation resistance of ACR/SBS asphalt. The modified asphalt (ACR/SBS-2) had good high- and low- temperature performances, as well as an appropriate viscosity and low softening point difference, as a research object of aging. After short-term aging, the changes in the high- and low-temperature performances and workability of ACR/SBS asphalt were reduced. Taking the softening point as the target performance, the softening point of ACR/SBS asphalt was less affected by aging time and temperature, indicating that ACR/SBS asphalt was not sensitive to aging temperature and had good stability and aging resistance. From the micrograph by microscope, it was found that ACR/SBS asphalt could maintain a relatively stable polyphase structure for aging resistance.

## 1. Introduction

With the rapid development of new solid waste utilization technology in the road industry, renewable materials-modified asphalts represented by waste crumb rubber powder have been widely used in the field of civil engineering [1,2,3]. Scholars have systematically studied the modification process, road performance, and application of waste crumb rubber powder-modified asphalt in hot mix asphalt mixtures [4,5,6,7,8]. In order to further improve the utilization efficiency of waste crumb rubber and improve the road performance of crumb rubber-modified asphalt, many scholars have studied the activation process of crumb rubber powder.

The commonly used crumb rubber powder activation methods include physical activation methods such as the microwave method based on the thermal environment and ultrasonic method [9], chemical activation methods using chemical adjuvants [9], and biological methods [10]. In addition to the above activation methods, the mechanochemical methods using chemical adjuvants combined with mechanical force are also applied to the activation of crumb rubber powder [11]. The mechanochemical method can effectively play the role of desulfurization activation of additives through rubber broken to increase the surface roughness and activity of rubber powder. The mechanochemical method has the advantages of simple equipment, high production efficiency, low cost, and weak smell, and is very suitable for large factory production, which was chosen in this study. Many researchers believe that the storage stability and workability of crumb rubber-modified asphalt prepared from rubber powder significantly improve after desulfurization activation [12,13]. Juganaru et al. observed that according to the micro-morphology of activated crumb rubber powder (ACR)-modified asphalt, the ACR was more evenly distributed in asphalt, and the viscosity of ACR-modified asphalt was significantly reduced [14]. Shatanawi et al. pointed out that the water stability and rutting resistance of the ACR-modified asphalt mixture were better than those of the ordinary crumb rubber-modified asphalt mixture [15]. Liu et al. used a wet process to prepare crumb rubber-modified asphalt with different ACR contents and optimized the preparation parameters of ACR based on viscosity and the softening point difference. The asphalt mixture containing a 60% content of ACR possessed optimal comprehensive properties in terms of high-temperature rutting resistance, cracking resistance, and moisture resistance [16]. Chen et al. studied a new treatment method of crumb rubber impact on the high-temperature rheological performances and found that ACR-modified asphalt had better rheological performances at high temperatures and short-term aging performances [17]. The above studies showed that ACR can improve some properties of crumb rubber-modified asphalt, but ACR-modified asphalt still has problems such as difficult construction and poor aging resistance.

As a widely used asphalt modifier, styrene-butadiene-styrene block copolymer (SBS) can be used with crumb rubber powder to combine the advantages of two kinds of asphalt modifier through composite modification to meet the higher performance requirements of asphalt pavement [18,19,20,21]. Zhang et al. found that adding SBS with the content of 3% (mass fraction) into crumb rubber-modified asphalt with a rubber content of 15% can improve the high- and low-temperature performance, and the toughness of composite-modified asphalt could be enhanced. Adding sulfur with the content of 0.2% to moderate the vulcanization of composite-modified asphalt can further improve its storage stability and flexibility [19]. Huang et al. studied the low-temperature properties of soluble rubber powder/SBS composite-modified asphalt by the low-temperature ductility test and bending beam rheological test. The results showed that when the SBS content is 2%, the low-temperature plastic deformation capacity and low-temperature rheological properties of composite-modified asphalt are improved. However, the higher addition of SBS or crumb rubber may weaken the improvement effect of low-temperature properties of crumb rubber-modified asphalt. When the SBS content is too high, the aromatic content in asphalt is excessively reduced, resulting in the decline in the low-temperature rheological properties of composite-modified asphalt [22,23]. Liang et al. prepared crumb rubber powder/SBS composite-modified asphalt by the high-speed shear process, and studied its linear viscoelasticity and storage stability. The results showed that compared with base asphalt, adding SBS and crumb rubber powder to asphalt can significantly improve its viscoelasticity and viscosity. When the SBS content exceeds 1%, it is feasible to use crumb rubber powder instead of some SBS as a modifier, and its modulus and viscoelasticity are significantly improved. However, the phase separation of composite-modified asphalt will occur during storage, and the storage stability still needs to be further improved [16]. Therefore, under the compound modification of crumb rubber powder and SBS, the high-temperature stability and aging resistance of composite-modified asphalt are significantly improved compared with SBS-modified asphalt. The relevant indexes of low-temperature crack resistance, viscosity toughness, and storage stability of composite-modified asphalt are higher than those of ordinary crumb rubber asphalt.

Crumb rubber powder/SBS composite-modified asphalt has been proven to improve the road performance of crumb rubber-modified asphalt and have good aging resistance. However, due to the change in physical and chemical properties before and after rubber powder activation, it is different from original crumb-rubber modified asphalt in the modification system, which will have an impact on the composite modification technology. Therefore, using ACR prepared by the mechanochemical method instead of original crumb rubber powder as a modifier, this study prepared modified asphalt samples with different modifiers and SBS contents. Conventional physical properties tests, a dynamic shear rheometer (DSR), and the thin-film oven test (TFOT) were used to study the conventional physical properties, high-temperature rheological properties, and aging resistance of asphalt. In addition, the action forms and distribution of modifiers in asphalt were observed by an optical microscope to characterize the micro-morphology of ACR/SBS composite-modified asphalt. The innovation of this study was to compare and analyze the influence of crumb rubber powder activation and the effect of SBS on crumb rubber powder-modified asphalt.

## 2. Materials and Methods

### 2.1. Experimental Raw Materials

The raw materials in this study included Maoming 70# road petroleum asphalt, ACR, and SBS, in which the ACR was obtained from crumb rubber powder with a size of 30 mesh by the mechanochemical method using a chemical adjuvant (organic disulfide, i.e., OD) combined with the mechanical force to accelerate the chemical reaction. Based on the previous studies [11,24,25], the activation process parameters of the mechanochemical method using the OD adjuvant (3% of crumb rubber powder by mass) were optimized as follows: the OD adjuvant content was 3%, the mixing temperature was 160 °C, and the mixing time was 30 min. A commercially available linear SBS was selected, and its main physical indexes are shown in Table 1.

### 2.2. Preparation of ACR/SBS Composite-Modified Asphalt

**Figure 1:**
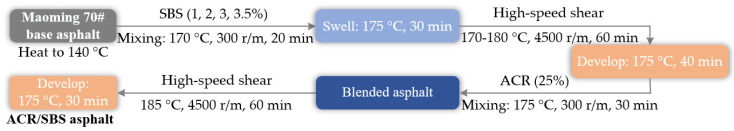
The preparation process of ACR/SBS-modified asphalt.

Based on the existing literature [11], the asphalt modified by untreated or activated crumb rubber powder (25% of base asphalt by mass) could be prepared, which are labeled as UCR asphalt or ACR asphalt, respectively. According to the previous process exploration, the following steps were adopted to prepare ACR/SBS composite-modified asphalt (as shown in Figure 1):

1. Place the base asphalt in an oven at 140 °C and heat it to the flowing state, and slowly add the weighed SBS (1%, 2%, 3%, and 3.5% of base asphalt by mass) with a mixing speed of 300 r/min. During the addition process, raise the temperature to 170 °C and maintain it for 20 min.
2. Put the mixed asphalt in an oven at 175 °C and swell for 30 min.
3. Place the swelled SBS asphalt into high-speed shear equipment, gradually increase the shear rate to about 4500 r/min, control the temperature at 170–180 °C, and then take it out after shearing for 1 h.
4. Put the modified asphalt in an oven at 175 °C and develop for 40 min.
5. Heat the developed SBS asphalt to the flowing state and slowly add the weighed ACR (25% of base asphalt by mass) with a mixing speed of 300 r/min. During the addition process, raise the temperature to 175 °C and maintain it for 30 min.
6. Place the blended asphalt into high-speed shear equipment, gradually increase the shear rate to about 4500 r/min, control the temperature at 185 °C, and then take it out after shearing for 1 h.
7. Put the prepared ACR/SBS asphalt into an oven at 175 °C for 30 min.

### 2.3. Experimental Scheme and Methods

**Figure 2:**
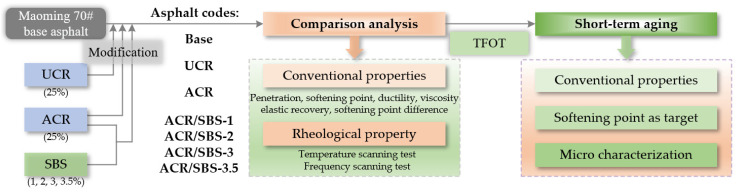
The flow chart of this study.

The asphalt samples were first prepared including seven asphalt codes, i.e., base, UCR, ACR, and ACR/SBS-1/2/3/3.5. Then, the asphalt samples could be used for the conventional physical properties test, high-temperature rheological test, thin-film oven test, and micro-characterization test including a stereoscopic microscope and Fourier infrared spectroscopy. The flow chart of this study is shown in Figure 2.

#### 2.3.1. Conventional Physical Properties Test

In this study, according to the Chinese standard “Standard Test Methods of Bitumen and Bituminous Mixtures for Highway Engineering” (JTG E20-2011) and previous studies [26,27], the penetration, softening point, ductility at 5 °C, viscosity, elastic recovery, and softening point difference (Δ) were tested for base asphalt, UCR asphalt, ACR asphalt, and ACR/SBS-1/2/3/3.5 asphalt.

#### 2.3.2. High-Temperature Rheological Test

The high-temperature rheological test of base asphalt, UCR asphalt, ACR asphalt, and ACR/SBS-1/2/3/3.5 asphalt was carried out by using a dynamic shear rheometer (DSR) [28]. In this study, the temperature scanning test in the temperature range of 52–82 °C was performed under the strain control mode with the strain value of *γ* = 12% and frequency of *ω* = 10 rad/s, and the frequency scanning test in the frequency range of 0.1 rad/s~100 rad/s was carried out under the strain control mode with the strain value of *γ* = 2% and temperature of 60 °C. By comparing and analyzing the rutting factor, complex shear modulus, and phase angle, the high-temperature properties of composite-modified asphalt could be characterized for different asphalt samples. Temperature scanning at the same frequency and different temperatures, and frequency scanning at the same temperature and different frequencies were conducted to explore the changes in asphalt viscoelastic parameters under two modes for the high-temperature performance from the perspective of temperature dependence and time dependence, respectively.

#### 2.3.3. Thin-Film Oven Test

In this study, the thin-film oven test (TFOT) was used to simulate the short-term aging behavior of asphalt in the process of storage, transportation, mixing, and paving. The prepared UCR asphalt, ACR asphalt, and ACR/SBS-2 asphalt were insulated for 5 h in a film oven at 163 °C for the short-term aging test. Then, the physical properties of asphalt samples before and after aging were tested to characterize the corresponding aging resistance by comparison and analysis.

In addition, the prepared UCR asphalt, ACR asphalt, and ACR/SBS-2 asphalt were insulated for different aging durations (5 h, 10 h, 15 h, and 20 h) in a film oven at different aging temperatures (150 °C, 163 °C, and 180 °C) for the short-term aging test. By comparing the variation in softening point of modified asphalt before and after aging, the effects of short-term aging factors (i.e., aging time and temperature) on the aging resistance of asphalt were studied with the softening point as the target performance.

## 3. Results and Discussion

### 3.1. Effect of ACR/SBS on the Conventional Physical Properties of Asphalt

**Figure 3:**
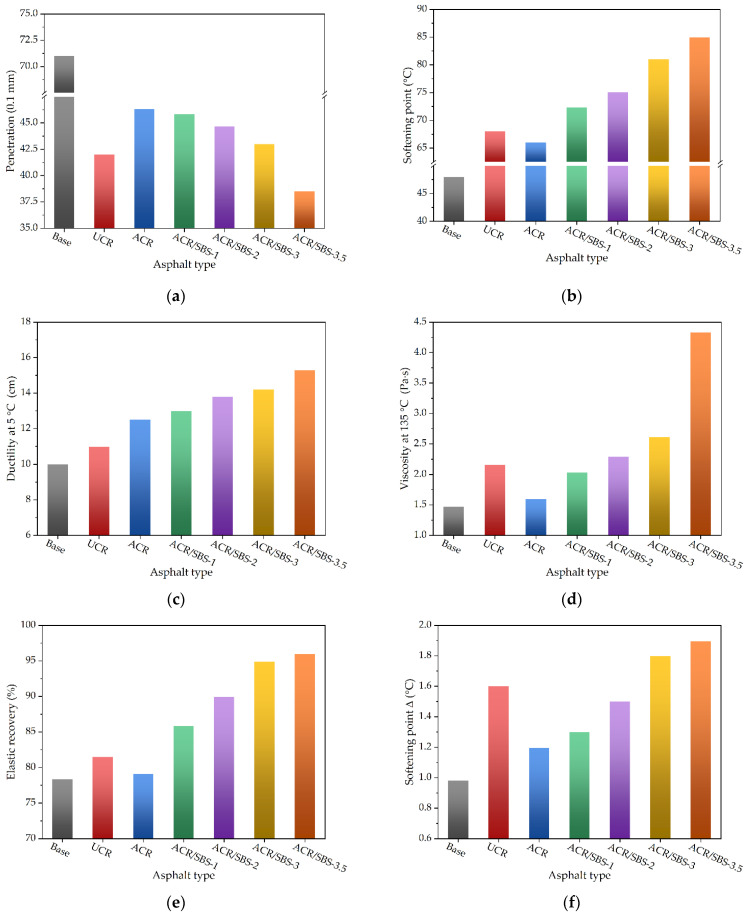
The conventional physical properties of different asphalt types: (**a**) penetration; (**b**) softening point; (**c**) ductility at 5 °C; (**d**) viscosity at 135 °C; (**e**) elastic recovery; (**f**) softening point Δ.

According to the above experimental scheme, the prepared base asphalt, UCR asphalt, ACR asphalt, and ACR/SBS-1/2/3/3.5 asphalt were tested to obtain the conventional physical properties, and the comparison results are shown in Figure 3.

From Figure 3a, it can be seen that the penetration of asphalt decreases by adding crumb rubber powder and SBS, and the penetration of ACR/SBS asphalt decreases with the increase in SBS content. Except for the SBS content being 3.5% (i.e., ACR/SBS-3.5 asphalt), the penetration of ACR/SBS asphalt is greater than that of UCR asphalt. In Figure 3b, the values of the softening point of asphalt modified by crumb rubber powder and SBS increase in comparison with base asphalt. It could also be found that with the increase in SBS content, the softening point of ACR/SBS asphalt gradually increases, and the softening point of ACR/SBS asphalt is higher than that of crumb rubber-modified asphalt.

In Figure 3c, it can be seen that with the addition of crumb rubber and SBS, the ductility of modified asphalt gradually increases, and the ductility of ACR/SBS asphalt increases with the SBS content. When the SBS content is 3.5%, the ductility of ACR/SBS-3.5 asphalt reaches more than 15 cm. As shown in Figure 3d, with the increase in SBS content, the viscosity of ACR/SBS asphalt increases gradually. When the SBS content is 2.0%, the viscosity of ACR/SBS-2 asphalt is basically the same as that of UCR asphalt.

It can be seen from Figure 3e that with the increase in SBS content, the elastic recovery rate of ACR/SBS asphalt gradually increases. When the SBS content is 3.5%, the elastic recovery rate of ACR/SBS-3.5 asphalt can reach 96%. At the same time, in Figure 3f, with the increase in SBS content, the softening point difference (Δ) of ACR/SBS asphalt also increases gradually, but it still meets the specification requirements. When the SBS content is less than 3%, the softening point difference (Δ) of ACR/SBS asphalt is less than that of UCR asphalt. According to the softening point difference (Δ) of UCR, it can be seen that one of the risks of using crumb rubber is the poor storage stability. Compared with UCR, the storage stability of ACR or ACR/SBS composite-modified asphalt improves due to the smaller softening point difference (Δ). However, when the SBS content is too high, the storage stability of ACR/SBS asphalt becomes worse.

The reason for the above phenomena is that the modification process of asphalt containing crumb rubber powder and SBS is mainly physical blending, which is also consistent with previous studies [13,29,30]. The addition of crumb rubber powder or SBS is equivalent to the increase in the reinforcing phase in the modified asphalt system. The performance variation trend of ACR/SBS asphalt is equivalent to the superposition of the performance trends of ACR-modified asphalt and SBS-modified asphalt. Thus, when the SBS content is less than 3%, the softening point and ductility of ACR/SBS asphalt improve at the same time, and the viscosity and penetration are more appropriate, enhancing the high-temperature performance. In addition, the elastic recovery performance of modified asphalt is also enhanced. However, when the SBS content is too high, the storage stability of modified asphalt becomes worse. Therefore, it is necessary to control the contents of ACR and SBS to reduce the segregation of modified asphalt.

### 3.2. Effect of ACR/SBS on the High-Temperature Rheological Properties of Asphalt

#### 3.2.1. Temperature Scanning Test

**Figure 4:**
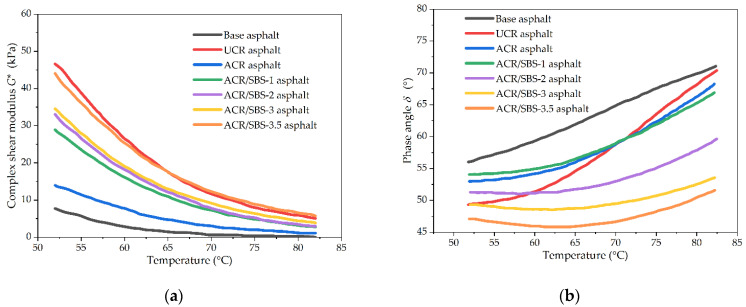
The temperature scanning test results of different asphalt types: (**a**) complex shear modulus; (**b**) phase angle.

The temperature scanning test was performed in the temperature range of 52–82 °C under the strain control mode with the strain value of *γ* = 12% and frequency of *ω* = 10 rad/s, and the comparison results of complex shear modulus and phase angle are shown in Figure 4. The complex shear modulus G* is used to characterize the deformation resistance, and the phase angle *δ* indicates the proportion and corresponding influence degree of elastic and viscous components of materials.

Figure 4a shows the relationship between the complex shear modulus of asphalt and temperature. It can be seen from Figure 4a that for the same asphalt type, the higher the test temperature, the smaller the complex shear modulus of asphalt. The complex shear modulus of asphalt modified by ACR or ACR/SBS is significantly higher than that of base asphalt, but lower than that of UCR asphalt. At the same temperature, with the increase in SBS content, the complex shear modulus of ACR/SBS asphalt gradually increases, but the difference between the complex shear modulus gradually decreases with the temperature. When the temperature is too high, the complex shear modulus of ACR/SBS asphalt is close to or higher than that of UCR asphalt. The addition of SBS greatly improves the complex shear modulus of ACR/SBS asphalt.

The relationship between the phase angle of asphalt and temperature is shown in Figure 4b. It can be seen that with the increase in temperature, the phase angle of ACR/SBS-2/3/3.5 asphalt decreases first and then increases. When the temperature is about 63 °C, the phase angle of these three ACR/SBS asphalt samples appears with the lowest value. However, the phase angle of other asphalt samples increases with the temperature. At the same temperature, the greater the SBS content, the smaller the phase angle of the corresponding ACR/SBS asphalt, and the better the high-temperature deformation resistance of ACR/SBS asphalt. When the test temperature reaches 80 °C, the phase angle of ACR/SBS asphalt is still less than 65°, which shows that the three types of asphalt have excellent high-temperature performance, and the presence of SBS has a significant effect on the phase angle of modified asphalt.

**Figure 5:**
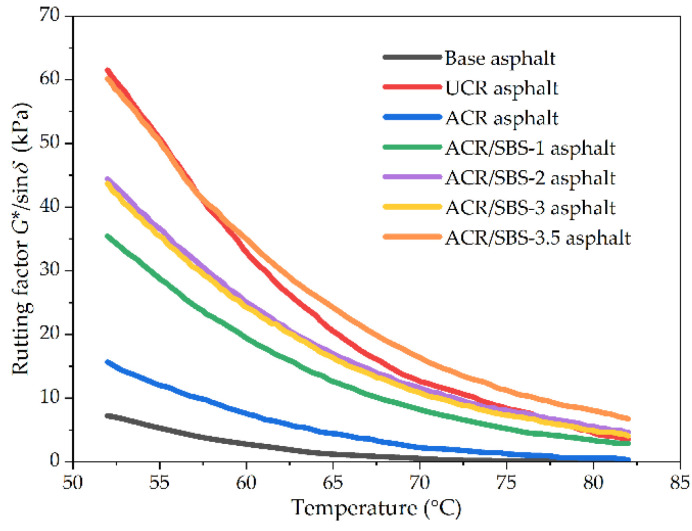
The rutting factor results of different asphalt types in the temperature scanning test.

In general, the rheological properties of asphalt cannot be fully reflected only by a single basic index of rheological properties (i.e., G* or *δ*). The rutting factor (G*/sin *δ*) is a new index derived from the basic index of rheological properties, which can characterize the ability of asphalt materials to resist permanent deformation at high temperature. Figure 5 shows the relationship between the rutting factor and temperature for asphalt samples. It can be seen from Figure 5 that the variation trends of the rutting factor with temperature and SBS content are consistent with the complex shear modulus for different asphalt types. Compared with ACR asphalt, the addition of SBS greatly improves the rutting factor of ACR/SBS asphalt. As an elastomer, SBS will form a network structure in asphalt, limiting the movement of crumb rubber powder particles. Thus, SBS plays the role of fixing crumb rubber powder particles, to significantly improve the high-temperature stability of modified asphalt.

#### 3.2.2. Frequency Scanning Test

**Figure 6:**
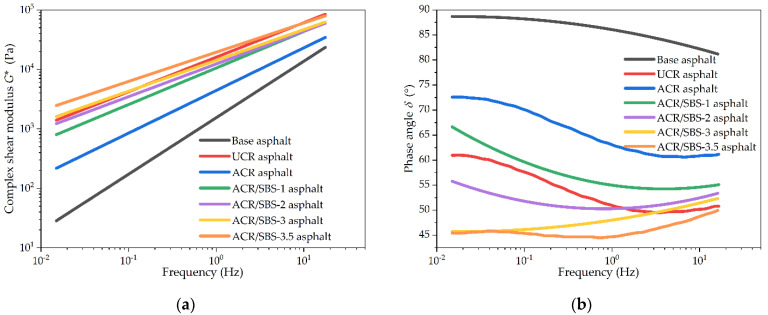
The frequency scanning test results of different asphalt types: (**a**) complex shear modulus; (**b**) phase angle.

The frequency scanning test was carried out in the frequency range of 0.1 rad/s~100 rad/s under the strain control mode with the strain value of *γ* = 2% and temperature of 60 °C. The comparison results of complex shear modulus and phase angle for different asphalt samples in the frequency scanning test are shown in Figure 6.

In Figure 6a, there are linear relationships between the complex shear modulus and frequency for different asphalt samples in double logarithmic coordinates. It can be seen from Figure 6a that compared with ACR asphalt, the complex shear modulus of ACR/SBS asphalt is greatly improved, and the complex shear modulus of ACR/SBS asphalt increases with the frequency. At the same frequency, with the increase in SBS content, the complex shear modulus of ACR/SBS asphalt gradually increases. The complex shear modulus of asphalt modified by ACR or ACR/SBS is also significantly higher than that of base asphalt. When the SBS content increases, the complex shear modulus of ACR/SBS asphalt is close to or higher than that of UCR asphalt.

The relationship between the phase angle and frequency for different asphalt samples in single-logarithmic coordinates is shown in Figure 6b. It can be seen that when the temperature is 60 °C, the phase angle of different asphalt samples presents a certain plateau period under low-frequency loading. With the increase in frequency, the phase angle of base asphalt and asphalt-modified crumb rubber particles decreases; however, the phase angle of ACR/SBS composite-modified asphalt first decreases and then increases. At the same frequency, the greater the SBS content, the smaller the phase angle of the corresponding ACR/SBS asphalt, and the better the high-temperature deformation resistance of ACR/SBS asphalt. When the test frequency reaches 10 Hz, the phase angle of ACR/SBS asphalt is less than 60°, which shows that ACR/SBS asphalt has excellent high-temperature performance. As a typical elastomer, SBS can effectively fill the lack of elastic components caused by desulfurization activation, and the phase angle of ACR/SBS composite-modified asphalt is greatly reduced. Thus, the presence of SBS has a significant effect on the phase angle of modified asphalt.

**Figure 7:**
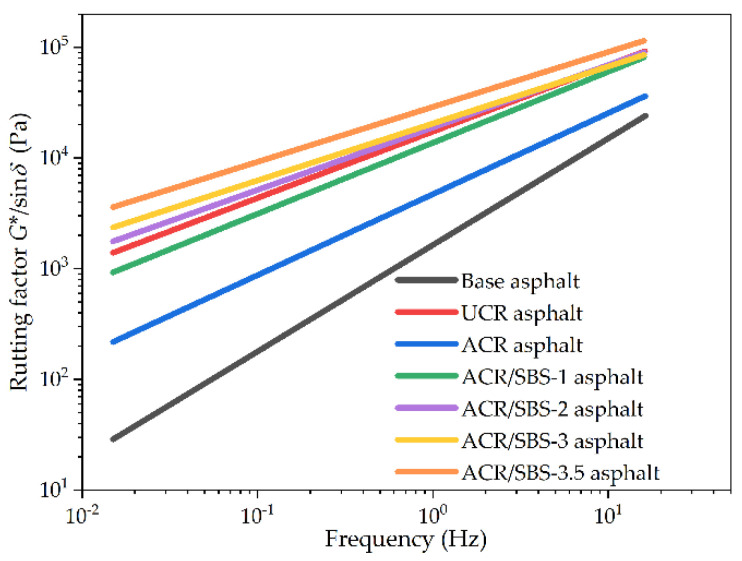
The rutting factor results of different asphalt types in the frequency scanning test.

The relationship results between the rutting factor and frequency for asphalt samples in the frequency scanning test are shown in Figure 7. As seen in Figure 7, similar to the complex shear modulus, the variation trend of the rutting factor with frequency and SBS content is consistent with that of the complex shear modulus for various asphalt types, and the variation relationship between the complex shear modulus and frequency is also linear in double logarithmic coordinates. The rutting factor of different asphalt samples increases with the frequency. The addition of SBS greatly improves the rutting factor of ACR/SBS asphalt compared with ACR asphalt, which could be beneficial to improve its high-temperature performance.

### 3.3. Effect of ACR/SBS on the Aging Resistance of Asphalt

**Figure 8:**
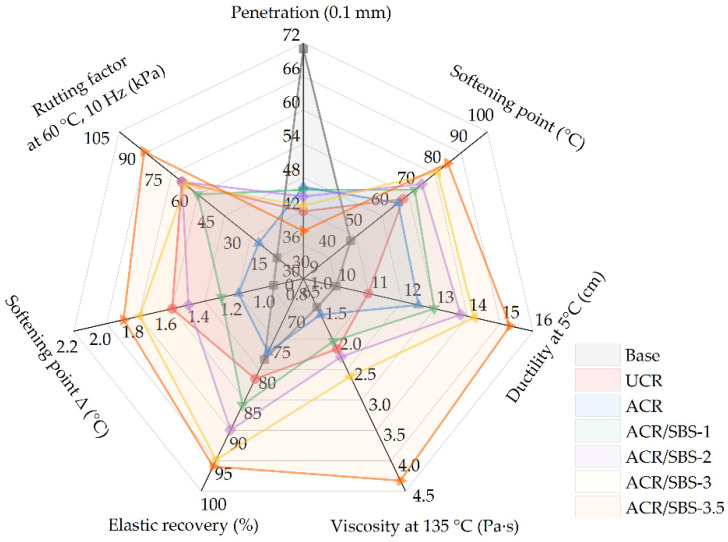
Radar chart based on conventional physical properties and high-temperature rheological properties of different asphalt types.

According to the comparison results including penetration, softening point, ductility at 5 °C, viscosity at 135 °C, elastic recovery, softening point Δ, and rutting factor in the radar chart (Figure 8), the addition of SBS improves the high- and low-temperature performances and elastic recovery performance of modified asphalt. However, when the SBS content is higher, the viscosity and storage stability of ACR/SBS asphalt become worse. Considering the indexes of high- and low-temperature performances, viscosity, and construction workability, ACR/SBS-2 asphalt has the best comprehensive performance, which not only has good high- and low-temperature performance, but also appropriate viscosity and low softening point difference. Then, the ACR (25% of base asphalt by mass) and SBS (2% of base asphalt by mass) composite-modified asphalt was selected as the research object used for the follow-up study.

#### 3.3.1. Conventional Physical Properties of Modified Asphalt under Short-Term Aging

**Figure 9:**
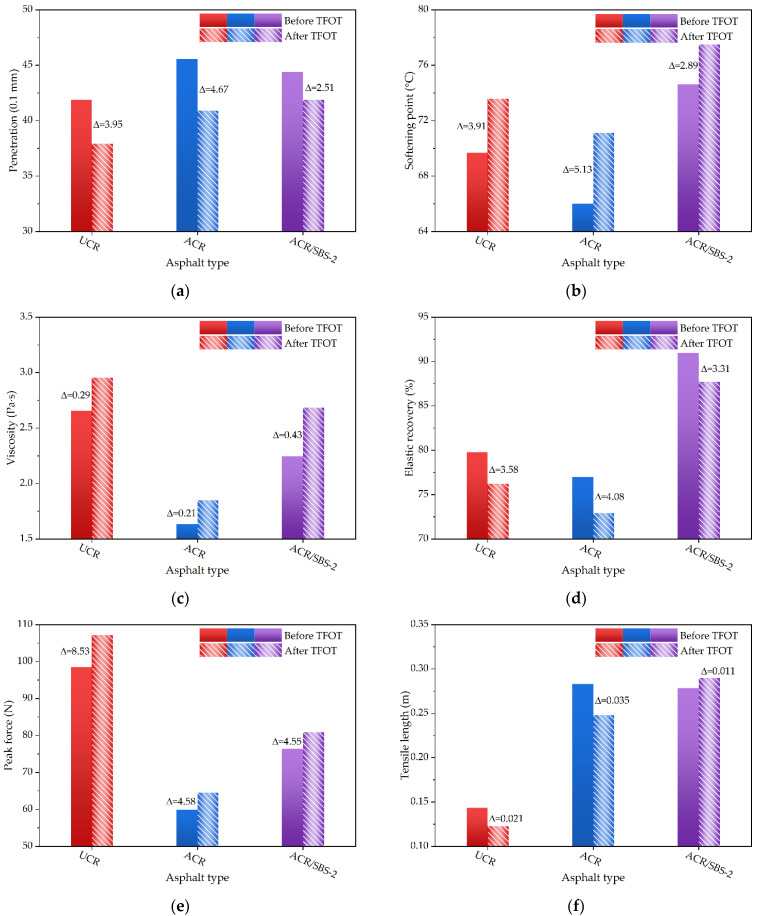
The conventional physical properties of different asphalt types: (**a**) penetration; (**b**) softening point; (**c**) viscosity; (**d**) elastic recovery; (**e**) peak force; (**f**) tensile length.

According to the penetration test of modified asphalt (i.e., UCR asphalt, ACR asphalt, and ACR/SBS-2 asphalt) before and after short-term aging, the effect of short-term aging on the viscosity of modified asphalt was analyzed, as shown in Figure 9a. It can be seen from Figure 9a that the penetration of the three modified asphalt samples after the TFOT decreases, among which the penetration reduction of ACR/SBS-2 asphalt is the least, and the penetration reduction of ACR asphalt is the most. This may be because under the action of thermal oxygen aging, the light components in asphalt volatilize, oxidize, and harden the intermolecular structure, which leads to the hardening of asphalt. Thus, the addition of SBS can reduce the hardening degree of modified asphalt.

The softening point variation of modified asphalt before and after short-term aging is used to analyze the effect of short-term aging on the high-temperature stability of modified asphalt, and Figure 9b shows the softening point test results. It can be seen that the softening point of the three modified asphalt samples increases in varying degrees after the TFOT, among which the softening point of ACR asphalt increases the most, and the softening point of ACR/SBS asphalt increases the least. After thermal oxygen aging, the light components in asphalt volatilize, oxidize, and harden the intermolecular structure, leading to a larger softening point value of asphalt. However, the aging process of crumb rubber-modified asphalt is more complex, in which, in addition to the aging of asphalt and crumb rubber powder, the interaction between crumb rubber powder and asphalt in the process of thermal oxygen aging should also be considered. The particle structure of ordinary crumb rubber powder is complete, so the light components that enter the internal network structure are not easy to volatilize, indicating that the thermal stability of ordinary crumb rubber-modified asphalt (i.e., UCR asphalt) is good. After desulfurization and activation of crumb rubber powder, the internal network structure of crumb rubber powder is destroyed. In the process of short-term aging, the light components of asphalt are easier to volatilize, resulting in a large increase in the softening point of ACR asphalt. On the other hand, the addition of SBS into ACR asphalt can more fully absorb the light components and reconstruct the network structure. Therefore, the thermal stability of ACR/SBS asphalt significantly improves.

The rotary viscosity test of modified asphalt before and after short-term aging was carried out to analyze the effect of short-term aging on the construction workability of modified asphalt, as shown in Figure 9c. As seen in Figure 9c, the viscosity of the three modified asphalts increases in varying degrees after the TFOT, in which the increase variation in viscosity of ACR asphalt is the least, and the increase variation in viscosity of ACR/SBS asphalt is larger. The viscosity values of three kinds of modified asphalt after the TFOT are still less than 3.5 Pa·s, which meets the specification requirements. Similarly, the light components in asphalt volatilize, oxidize, and harden the intermolecular structure, leading to the viscosity increasing of modified asphalt. Through the viscosity between UCR asphalt and ACR asphalt, the internal network structure of crumb rubber powder is damaged after activation treatment, resulting in the viscosity of ACR asphalt being lower than that of UCR asphalt. With the progression of short-term aging, the viscosity of ACR asphalt still increases slightly. In addition, due to the larger average particle size of the SBS modifier and smaller average particle size of ACR, the viscosity of ACR asphalt before and after aging is smaller, while the viscosity of ACR/SBS asphalt is larger.

The elastic recovery test of modified asphalt before and after short-term aging was conducted to analyze the effect of short-term aging on the elastic recovery performance of modified asphalt, as shown in Figure 9d. The elastic recovery rate of ACR asphalt is lower than that of UCR asphalt, but the addition of SBS greatly improves the elastic recovery rate of modified asphalt. After short-term aging, the elastic recovery rate of modified asphalt decreases in varying degrees, among which the elastic recovery rate of ACR asphalt decreases the most, and the elastic recovery rate of ACR/SBS asphalt decreases the least. The elastic recovery ability of crumb rubber-modified asphalt mainly depends on the elasticity of crumb rubber powder particles. Therefore, the swelling and degradation degree of crumb rubber powder in asphalt play a major role in its elastic recovery ability. After desulfurization and activation, the internal network structure of crumb rubber powder is damaged and becomes smaller, and crumb rubber powder further loses elasticity and reduces its elastic recovery rate. After adding SBS, the internal network structure of modified asphalt is reconstructed, and the elastic recovery performance of ACR/SBS asphalt improves.

The force ductility test of modified asphalt before and after short-term aging was performed to analyze the effect of short-term aging on the low-temperature crack resistance of modified asphalt, as shown in Figure 9e,f. The peak force of the three modified asphalts increases after short-term aging. The light components in crumb rubber-modified asphalt volatilize, resulting in the hardening of modified asphalt and the increase in peak force. The tensile length of UCR asphalt and ACR asphalt decreases, and the tensile length of ACR/SBS asphalt increases slightly. During short-term aging, asphalt hardens by oxidation, and the modifier continues to swell and crack slowly. The pyrolysis products of crumb rubber powder and SBS can effectively make up for the adverse effects caused by the volatilization and oxidation of light components in asphalt. Therefore, the tensile length and toughness of ACR/SBS asphalt improve.

#### 3.3.2. Short-Term Aging Factors with Softening Point as Target Performance

**Figure 10:**
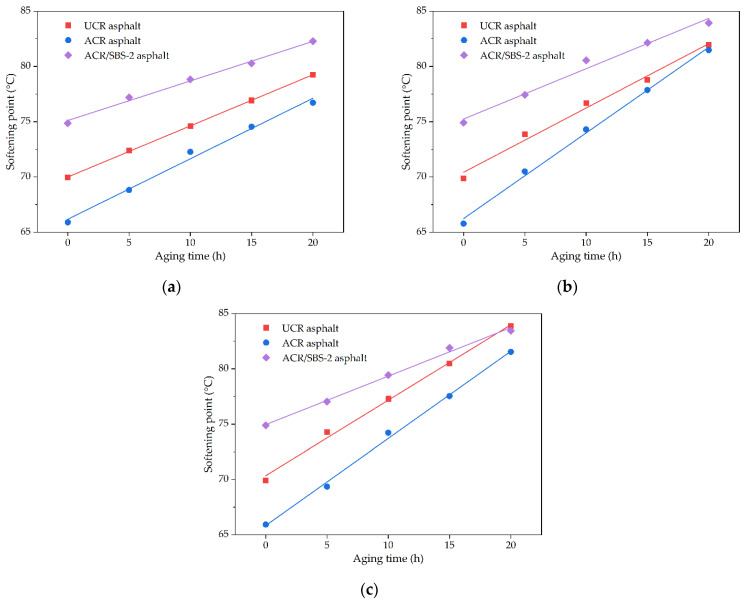
The softening point results of different asphalt types under different aging conditions: (**a**) 150 °C; (**b**) 163 °C; (**c**) 180 °C.

The short-term aging test was carried out under different aging durations (5 h, 10 h, 15 h, and 20 h) in a film oven at different aging temperatures (150 °C, 163 °C, and 180 °C) for UCR asphalt, ACR asphalt, and ACR/SBS-2 asphalt. By comparing the variation in softening point of modified asphalt under different aging conditions, the effect of aging time and temperature on the aging resistance of modified asphalt was studied with the softening point as the target performance, as shown in Figure 10.

It can be seen that the softening points of modified asphalt increase linearly with aging time at different aging temperatures. Meanwhile, at the same aging temperature, the growth range of the softening point of different modified asphalt samples is significantly different. When the aging temperature is 150 °C, the linear curve slope of the softening point versus aging time for ACR/SBS-2 asphalt is the smallest, and the slope of ACR asphalt is the largest, indicating that ACR/SBS-2 asphalt has the best aging resistance at the aging temperature of 150 °C. At the same time, there is a small difference between the curve slopes of these three modified asphalts at the aging temperature of 150 °C.

For the same modified asphalt, the growth range of the softening point versus aging time is different at different aging temperatures. The linear curve slope of the softening point versus aging time for ACR/SBS-2 asphalt changes little with the aging temperature, while the slope of ACR asphalt changes the most, indicating that ACR/SBS-2 asphalt is not sensitive to the aging temperature and has good short-term aging resistance. When the aging temperature is lower, crumb rubber powder and SBS in modified asphalt basically do not change, while the light components in base asphalt volatilize and oxidize. Therefore, the softening point of modified asphalt increases with the aging time, and the growth range difference of the softening point is less for these three kinds of modified asphalt. When the aging temperature is higher, the modifier in modified asphalt degrades and desulfurizes. These depolymerized and desulfurized components can make up for the adverse effects of asphalt aging, and the degradation of SBS is more obvious. Therefore, the softening point of ACR/SBS-2 asphalt increases slightly with aging time, and its aging resistance is better.

### 3.4. Micro-Characterization of ACR/SBS Composite-Modified Asphalt

**Figure 11:**
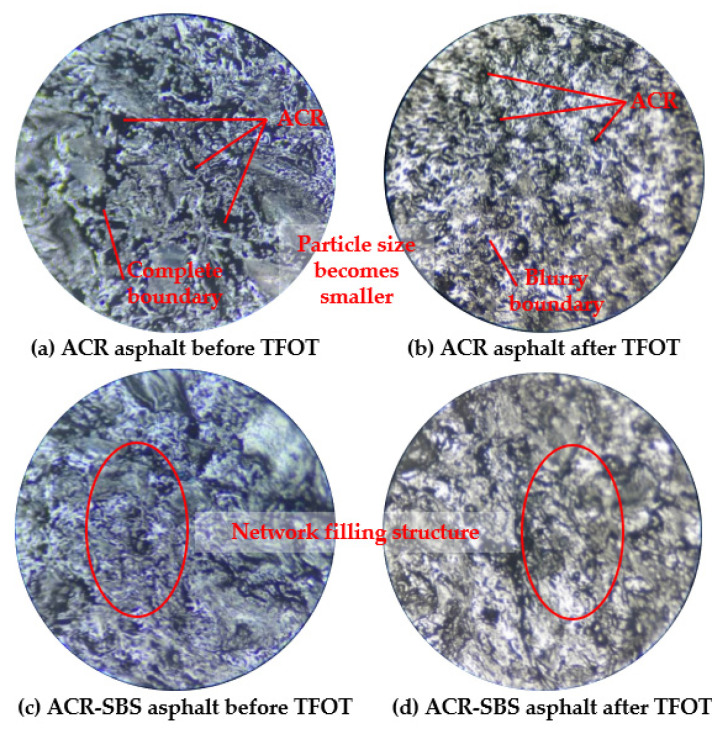
The 80× micrograph of ACR asphalt and ACR/SBS-12 asphalt before and after TFOT: (**a**) ACR asphalt before TFOT; (**b**) ACR asphalt after TFOT; (**c**) ACR/SBS-12 asphalt before TFOT; (**d**) ACR/SBS-12 asphalt after TFOT.

The asphalt sample sections of ACR asphalt and ACR/SBS-2 asphalt before and after short-term aging were magnified by 80 times with an optical microscope, and are shown in Figure 11.

It can be seen from Figure 11a that the particle sizes of ACR are different, which are more evenly dispersed in asphalt, and there is an obvious boundary between ACR and asphalt. ACR particles with smaller particle size could form a discontinuous network structure with asphalt, and ACR particles with larger particle size would disperse in asphalt and play a filling role. It can be seen from Figure 11b that after short-term aging of ACR asphalt, the boundary between ACR particles and asphalt becomes blurred, and the ACR particle size becomes smaller, in which their dispersion is more uniform, and discontinuous network structures would be formed in asphalt. During the short-term aging process, ACR particles are partially degraded, and the degradation products are distributed in asphalt in the form close to the original components of asphalt such as resin and asphaltene, making them easier for ACR and asphalt to form a network structure.

In Figure 11c, after the composite modification of ACR and SBS, SBS particles and ACR with smaller particle size could form an interpenetrating network, and ACR with larger particle size are filled in the network. A network filling structure composed of asphalt, SBS, and ACR with different particle sizes is formed inside ACR/SBS-2 asphalt, and the existence of the network structure improves the compatibility and stability of the heterogeneous structure. As can be seen from Figure 11d, the structure of ACR/SBS-2 asphalt remains relatively complete, and the boundary between ACR and asphalt becomes blurred. However, due to the network structure formed by SBS, some ACR with large particle size does not undergo desulfurization and degradation, and their dispersion in the network structure would play a filling role. During the short-term aging process, the light components in base asphalt undergo condensation reaction to form resin and asphaltene. The activity of resin and asphaltene obtained by condensation reaction is higher than that of the original components. Because ACR and SBS will undergo desulfurization and degradation reaction under heating and oxygen conditions, the desulfurization degradation products could react with resin and asphaltene with strong activity to produce resin, asphaltene, and toluene-insoluble substances different from the original components of asphalt. Therefore, ACR/SBS-2 asphalt has excellent anti-aging properties, and ACR and SBS could play an anti-aging role.

## 4. Conclusions

In this study, the conventional physical properties, high-temperature rheological properties, and aging resistance of ACR/SBS composite-modified asphalt were investigated based on conventional physical properties tests, DSR, and TFOT. In addition, the action forms and distribution of modifiers in asphalt were observed by an optical microscope to characterize the micro-morphology. From the test results, the following conclusion could be drawn:

(1) After adding SBS, the softening point, ductility, and elastic recovery of ACR/SBS asphalt could be significantly improved, but the viscosity and softening point difference were also larger with the increase in SBS content. The complex shear modulus and rutting factor of ACR/SBS asphalt were greatly improved, while the phase angle was significantly reduced, indicating that SBS can effectively improve the high-temperature deformation resistance.

(2) The changes in high- and low-temperature performances and workability of ACR/SBS asphalt were reduced after short-term aging, and the addition of SBS could improve the aging resistance of modified asphalt. The softening point as the target performance of ACR/SBS asphalt was less affected by aging time and temperature, indicating that ACR/SBS asphalt was not sensitive to aging temperature with good stability and aging resistance.

(3) From the micrograph by microscope, ACR/SBS asphalt could maintain a relatively stable polyphase structure in the short-term aging process, which helped the degradation products of crumb rubber powder and SBS supplement the components in asphalt for aging resistance.

## Figures and Tables

**Table 1:** The basic physical properties of SBS.

Structure Type	Block Ratio	Tensile Rate (%)	Tensile Strength (MPa)	Melt Index (g/10 min)	Density (g/cm^3^)
Linear	30/70	800	25	0.6	0.93

## Data Availability

The data presented in this study are available on request from the corresponding author.

## References

[1] Picado-Santos L.G., Capitao S.D., Neves J.M.C. (2020). Crumb rubber asphalt mixtures: A literature review. Constr. Build. Mater..

[2] Ma T., Wang H., He L., Zhao Y.L., Huang X.M., Chen J. (2017). Property characterization of asphalt binders and mixtures modified by different crumb rubbers. J. Mater. Civ. Eng..

[3] Saberi K.F., Fakhri M., Azami A. (2017). Evaluation of warm mix asphalt mixtures containing reclaimed asphalt pavement and crumb rubber. J. Clean. Prod..

[4] Riekstins A., Baumanis J., Barbars J. (2021). Laboratory investigation of crumb rubber in dense graded asphalt by wet and dry processes. Constr. Build. Mater..

[5] Ameli A., Babagoli R., Asadi S., Norouzi N. (2021). Investigation of the performance properties of asphalt binders and mixtures modified by crumb rubber and gilsonite. Constr. Build. Mater..

[6] Wang Q.Z., Wang N.N., Tseng M.L., Huang Y.M., Li N.L. (2020). Waste tire recycling assessment: Road application potential and carbon emissions reduction analysis of crumb rubber modified asphalt in china. J. Clean. Prod..

[7] Gao J., Yang J.G., Yu D., Jiang Y., Ruan K.G., Tao W.J., Sun C., Luo L.H. (2021). Reducing the variability of multi-source reclaimed asphalt pavement materials: A practice in china. Constr. Build. Mater..

[8] Gao J., Yao Y., Song L., Xu J., Yang J. (2022). Determining the maximum permissible content of recycled asphalt pavement stockpile in plant hot-mix recycled asphalt mixtures considering homogeneity: A case study in china. Case Stud. Constr. Mater..

[9] Xu M.Z., Liu J.J., Li W.Z., Duan W.F. (2015). Novel method to prepare activated crumb rubber used for synthesis of activated crumb rubber modified asphalt. J. Mater. Civ. Eng..

[10] Yu R.B., Gong Z.H., Guo W.H., Zhang H.B., Liu C.L. (2016). A novel grafting-modified waste rubber powder as filler in natural rubber vulcanizates. J. Appl. Polym. Sci..

[11] Zhang H.G., Zhang Y.P., Chen J., Liu W.C., Wang W.S. (2022). Effect of desulfurization process variables on the properties of crumb rubber modified asphalt. Polymers.

[12] Kedarisetty S., Biligiri K.P., Sousa J.B. (2016). Advanced rheological characterization of reacted and activated rubber (rar) modified asphalt binders. Constr. Build. Mater..

[13] Lv S.T., Ma W.B., Zhao Z.G., Guo S.C. (2021). Improvement on the high-temperature stability and anti-aging performance of the rubberized asphalt binder with the lucobit additive. Constr. Build. Mater..

[14] Juganaru T., Bombos M., Vasilievici G., Bombos D. (2015). Devulcanized rubber for bitumen modification. Mater. Plast..

[15] Shatanawi K.M., Biro S., Naser M., Amirkhanian S.N. (2013). Improving the rheological properties of crumb rubber modified binder using hydrogen peroxide. Road Mater. Pavement Des..

[16] Liu Q., Liu J.Z., Yu B., Zhang J.P., Pei J.Z. (2022). Evaluation and optimization of asphalt binder and mixture modified with high activated crumb rubber content. Constr. Build. Mater..

[17] Chen Z.X., Pei J.Z., Wang T., Amirkhanian S. (2019). High temperature rheological characteristics of activated crumb rubber modified asphalts. Constr. Build. Mater..

[18] Rasool R.T., Song P., Wang S.F. (2018). Thermal analysis on the interactions among asphalt modified with sbs and different degraded tire rubber. Constr. Build. Mater..

[19] Zhang F., Hu C.B. (2015). The research for structural characteristics and modification mechanism of crumb rubber compound modified asphalts. Constr. Build. Mater..

[20] Wang W.S., Tan G.J., Liang C.Y., Wang Y., Cheng Y.C. (2020). Study on viscoelastic properties of asphalt mixtures incorporating sbs polymer and basalt fiber under freeze-thaw cycles. Polymers.

[21] Song L., Zhang G.Q., Xie H.F., Gao J. (2022). Laboratory study on cr/sbs modified asphalt: Preparation and performance characterization. J. Renew. Mater..

[22] Tang N.P., Lv Q., Huang W.D., Lin P., Yan C.Q. (2019). Chemical and rheological evaluation of aging characteristics of terminal blend rubberized asphalt binder. Constr. Build. Mater..

[23] Yan C., Lv Q., Zhang A.A., Ai C., Huang W., Ren D. (2022). Modeling the modulus of bitumen/sbs composite at different temperatures based on kinetic models. Compos. Sci. Technol..

[24] Zhang B., Chen H.X., Zhang H.G., Kuang D.L., Wu J.Y., Zhang X.L. (2019). A study on physical and rheological properties of rubberized bitumen modified by different methods. Materials.

[25] Zhang B., Chen H.X., Zhang H.G., Wu Y.C., Kuang D.L., Guo F.J. (2020). Laboratory investigation of aging resistance for rubberized bitumen modified by using microwave activation crumb rubber and different modifiers. Materials.

[26] Wang W.S., Cheng Y.C., Chen H.P., Tan G.J., Lv Z.H., Bai Y.S. (2019). Study on the performances of waste crumb rubber modified asphalt mixture with eco-friendly diatomite and basalt fiber. Sustainability.

[27] Wang W.S., Cheng Y.C., Tan G.J., Liu Z.Y., Shi C.L. (2018). Laboratory investigation on high- and low-temperature performances of asphalt mastics modified by waste oil shale ash. J. Mater. Cycles Waste.

[28] Zhang L.M., Gao X.K., Wang W.S., Wang H., Zheng K.K. (2021). Laboratory evaluation of rheological properties of asphalt binder modified by nano-tio2/caco3. Adv. Mater. Sci. Eng..

[29] Chen T., Ma T., Huang X.M., Guan Y.S., Zhang Z.X., Tang F.L. (2019). The performance of hot-recycling asphalt binder containing crumb rubber modified asphalt based on physiochemical and rheological measurements. Constr. Build. Mater..

[30] Zhang J.W., Chen M.Z., Wu S.P., Zhou X.X., Zhao G.Y., Zhao Y.C., Cheng M. (2021). Evaluation of vocs inhibited effects and rheological properties of asphalt with high-content waste rubber powder. Constr. Build. Mater..

